# Kalman-Filter-Based Orientation Determination Using Inertial/Magnetic Sensors: Observability Analysis and Performance Evaluation

**DOI:** 10.3390/s111009182

**Published:** 2011-09-27

**Authors:** Angelo Maria Sabatini

**Affiliations:** The BioRobotics Institute, Scuola Superiore Sant’Anna, Piazza Martiri della Libertà 33, 56124 Pisa, Italy; E-Mail: sabatini@sssup.it; Tel.: +39-050-883-415; Fax: +39-050-883-101

**Keywords:** ambulatory human motion tracking, orientation determination, inertial measurement unit, Extended Kalman filter, Lie derivatives, observability of nonlinear systems

## Abstract

In this paper we present a quaternion-based Extended Kalman Filter (EKF) for estimating the three-dimensional orientation of a rigid body. The EKF exploits the measurements from an Inertial Measurement Unit (IMU) that is integrated with a tri-axial magnetic sensor. Magnetic disturbances and gyro bias errors are modeled and compensated by including them in the filter state vector. We employ the observability rank criterion based on Lie derivatives to verify the conditions under which the nonlinear system that describes the process of motion tracking by the IMU is observable, namely it may provide sufficient information for performing the estimation task with bounded estimation errors. The observability conditions are that the magnetic field, perturbed by first-order Gauss-Markov magnetic variations, and the gravity vector are not collinear and that the IMU is subject to some angular motions. Computer simulations and experimental testing are presented to evaluate the algorithm performance, including when the observability conditions are critical.

## 1. Introduction

Accurate tracking of the orientation of rigid bodies moving in a three-dimensional (3D) space is important in several applications, including navigation of man-made vehicles, robotics, machine interaction and, of main interest to us in this paper, ambulatory human motion analysis. Several approaches are available to create trackers that determine orientation based on measurements from, e.g., acoustic, mechanical, optical, inertial and magnetic sensors [1]. One popular approach is based on the principles behind inertial/magnetic sensing.

A state-of-the-art Inertial Measurement Unit (IMU) consists of a tri-axial accelerometer, a tri-axial gyro, and a tri-axial magnetic sensor, henceforth referred to as an integrated IMU. The 3D orientation can be computed by time-integrating the gyro output from known initial conditions. Due to low-frequency gyro bias drift this operation is subject to errors that tend to grow unbounded over time. On the other hand, gyros may help achieving accurate orientation estimates for highly dynamic motions. The initial conditions needed for gyro integration are usually given by the accelerometer used in combination with the magnetic sensor. The accelerometer can provide drift-free inclination estimates by sensing the gravity vector; the magnetic sensor, which would sense the earth magnetic field vector, helps providing drift-free heading estimates. Serious limitations affect their operation when applied to ambulatory human motion tracking [2–4]. First, the difficulty of correctly interpreting the acceleration signals exists, when the component due to the gravity field (vertical reference) coexists with the component related to the body motion [5]. Second, ferromagnetic materials nearby the IMU are critically disturbing sources when the magnetic sensor output is used to build the horizontal reference for heading estimation [6,7].

Accurate estimates of the three-dimensional (3D) orientation of a rigid body by inertial/magnetic sensing require that the complementary properties of gyros, accelerometers and magnetic sensors are exploited [8]. Sensor fusion techniques are needed in order that the aiding sensors (accelerometer and magnetic sensor) help mitigating the low-frequency gyro bias errors, while, in turn, the signals from the aiding sensors, which are prone to relatively high-frequency errors, are smoothed using gyro data. Different approaches are available to design sensor fusion algorithms [8]. In [9] a simple time-domain first-order complementary filter is designed: it performs low-pass filtering on the signals from the accelerometer-magnetic sensor pair and high-pass filtering on the signals from the gyro. Another possible approach is represented by the use of deterministic single-frame estimation techniques [8]. They all are based on the concept of vector matching, which requires, in principle, the measurements of constant reference vectors (e.g., gravity and the Earth’s magnetic field) [10]. Mostly, these techniques have been employed in gyro-free systems for tracking static or slowly moving bodies in environments containing only small magnetic sources [3,4,11]. In their original formulation, they are unable to provide sequential estimates of a time-varying orientation and of other parameters than the orientation, such as sensor biases [12]. In contrast with the previous approaches, which bypass at all statistical modeling in the estimator design, extended stochastic linear estimation techniques use a model for predicting aspects of the time behavior of a system (dynamic model) and a model of the sensor measurements (measurement model). There appears to be wide consensus that the Kalman filter is “perhaps *the* perfect tool for elegantly combining multisensory fusion, filtering, and motion prediction in a single fast and accurate framework” [13]. Since orientation determination is intrinsically a non-linear problem, Extended Kalman filters (EKFs) are the tools to work with [14].

In [15] an indirect-state formulation of the EKF for an inertial head-tracker is described; it operates on errors in the primary variables of Euler angles, angular velocity and gyro bias. The indirect-state formulation is chosen to provide fast response, *i.e.*, low latency due to computational demands of the algorithm. However, no expedient is provided to guard against the effects of body accelerations and magnetic disturbances. Very interesting is the work in [16], where the authors use one tri-axial accelerometer to measure inclination during dynamic tasks without requiring additional sensors, be they gyros or magnetic sensors. A KF-based algorithm is designed to estimate the different acceleration components, namely gravity and inertial acceleration, plus the accelerometer bias, based on few reasonable assumptions about the properties of human motion. In a later paper by the same group, an integrated IMU is used to estimate the full orientation matrix [17]. The KF for body segment orientation in [16] is thus extended with models of gyro and magnetic sensor biases, in the attempt to prevent heading drift and to compensate magnetic disturbances. A direct-state EKF is developed in [18], where the quaternion and the angular velocity are included in the vector state. Dynamic models of human limb motion in terms of first-order Gauss-Markov stochastic processes are used in developing the filter equations. The linear measurement equations include the components related to gyro measurements and components that are formulated in the quaternion space; this latter part of the measurement equations uses a deterministic single-frame estimation technique, *i.e.*, a Gaussian Newton optimizer, that computes the corresponding quaternion for each set of accelerometer and magnetic sensor measurements. No compensation of sensor errors or protection against magnetic effects is provided in this work. A direct-state quaternion-based formulation of the EKF is developed in [2], where angular velocity is considered a control input and active compensation (gyro bias and magnetic effects) is achieved by using state-augmentation techniques. Since the angular velocity is not part of the state vector, models of human motion dynamics are not necessary in the development of the filter equations [19]. The non-linear measurement equations are formulated by rotating the reference vectors in the body-frame using the estimated orientation matrix. The EKF is made adaptive by introducing vector selection schemes that work on the measured gravity and magnetic field vectors [4,20,21]: the measurement noise variances are increased in value, thus giving more weight to the filter predictions, when variations are found between the measured acceleration and the gravity, or between the measured magnetic field and the earth magnetic field.

The use of state-augmentation techniques is the preferred approach within the KF framework to take sensor errors and magnetic disturbances into account. At one point or another, however, the dimension of the state vector may create problems of system observability. Issues of system observability have sometimes been addressed, e.g., [21,22], without a formal analysis being carried out. A primary contribution of this paper is to elucidate under which conditions a KF-based algorithm for orientation determination using an integrated IMU is observable; in other words, the conditions when sufficient information is available for estimating a state vector that includes, in the present case, the quaternion of rotation, the magnetic variation superimposed to the magnetic reference vector and the gyro bias vector. To the best of our knowledge, this is the first time an observability analysis based on tools from the nonlinear control theory, namely the Lie derivatives, is performed in this regard. We take inspiration from the work described in [23], where the observability analysis is applied to a problem of IMU-camera calibration. The observability conditions are that the magnetic field vector, perturbed by first-order Gauss-Markov magnetic variations, and the gravity vector are not collinear, and that the IMU is subject to some angular motions. Computer simulations and experimental testing are presented to evaluate the algorithm performance, including when the observability conditions are critical.

## 2. Theoretical Background

### 2.1. Using Quaternions to Represent the Orientation

The 3D orientation of a rigid body is represented using two right-handed coordinate systems: the earth-fixed frame **E** = {e_1_ e_2_ e_3_} and the body-fixed frame $\text{B}=\left\{{{\text{e}}^{\prime }}_{1}\;{{\text{e}}^{\prime }}_{2}\;{{\text{e}}^{\prime }}_{3}\right\}$. In applications of motion tracking the motion of the E-frame due to the earth’s rotation is usually negligible; the E-frame is therefore an inertial frame. In the following, we also assume that the sensitivity axes of the IMU sensors are aligned with the axes of the B-frame, and the physical location of the accelerometer is where the origin of the B-frame is located, *i.e.*, no centripetal acceleration effects exist.

An arbitrary vector **x** in the 3D space can be represented in terms of the coordinates (or components) with respect to either of the frames:

(1) $${\text{x}}_{ℬ}={}_{ℰ}^{ℬ}\text{C}\;{\text{x}}_{ℰ}.$$

The subscripts E, B denote in which frame the vector **x** is represented. The columns of the orientation matrix ${}_{ℰ}^{ℬ}\text{C}$ are the representations of the **e***_i_*, *i* = 1,…,3 with respect to B, while its rows are the representations of the ${{\text{e}}^{\prime }}_{i}$, *i* = 1,..., 3 with respect to E. The orientation matrix and its transpose allow moving vector representations from (to) E to (from) B, respectively. The orientation matrix is a 3 × 3 orthogonal matrix with unit determinant, which belongs to the 3D special orthogonal group SO(3) of rotation matrices. The quaternion is a more parsimonious representation of orientation than the orientation matrix [24]; it is derived by formulating the orientation matrix as a homogeneous quadratic function of the Euler symmetric parameters *q_i_*, *i* = 1,...,4:

(2) $${}_{ℰ}^{ℬ}\text{C}(\bar{\text{q}})={\text{I}}_{3\times 3}-2q_{4}\;[\text{q}\times ]+2{\left[\text{q}\times \right]}^{2},$$

where the operator:

(3) $$[\text{q}\times ]=\left[\begin{array}{ccc} 0 & -q_{3} & q_{2}\\ q_{3} & 0 & -q_{1}\\ -q_{2} & q_{1} & 0 \end{array}\right]$$

is the standard vector cross product and **I**_*n*×*n*_ is the *n* × *n* identity matrix. It is commonplace to refer to **q** as the vector part and to *q*_4_ as the scalar part of the quaternion $\bar{\text{q}}={\left[{\text{q}}^{T}\;q_{4}\right]}^{T}$ :

(4) $$\text{q}={\left[q_{1}\;q_{2}\;q_{3}\right]}^{\;T}=\text{sin}(\theta /2){\text{n}}^{T},\;q_{4}=\text{cos}(\theta /2).$$

Here, the axis and angle of rotation (**n**,*θ*) are another valid representation of the orientation, closely related to the rotation vector **θ** = *θ* **n** [24]. As implied by Equation (4), in order to represent a valid rotation the quaternion must comply with the normalization constraint:

(5) $$\left|\bar{\text{q}}\right|=\sqrt{\sum_{i=1}^{4}q_{i}^{2}}=\sqrt{{\left|\text{q}\right|}^{2}+q_{4}^{2}}=1.$$

In the quaternion space the multiplication between two generic quaternions **q̄** and **q̄**′ is defined as follows:

(6) $$\bar{\text{q}}⊗{\bar{\text{q}}}^{\prime }={\left[{\left(q_{4}^{\prime }\text{q}+q_{4}{\text{q}}^{\prime }+[\text{q}\times ]\;\;{\text{q}}^{\prime }\right)}^{T}\;q_{4}q_{4}^{\prime }-\text{q}\cdot {\text{q}}^{\prime }\right]}^{\;T},$$

where the symbol · denotes the standard vector dot product.

Given the vector quaternion ${\bar{\text{x}}}_{ℰ}={\left[{\text{x}}_{ℰ}^{T}\;0\right]}^{T}$, namely a quaternion with null scalar part associated with the vector **x**_ℰ_, the vector quaternion:

(7) $${\bar{\text{x}}}_{ℬ}={\left[{\text{x}}_{ℬ}^{T}\;0\right]}^{T}={\bar{\text{q}}}^{-1}⊗{\bar{\text{x}}}_{ℰ}⊗\bar{\text{q}}$$

is associated with the vector **x**_ℬ_, rotated about the **n**-axis through an angle *θ*, see Equation (4). The inverse quaternion **q̄**^−1^ in Equation (7) is given by:

(8) $${\bar{\text{q}}}^{-1}⊗\bar{\text{q}}={\left[0^{T}\;1\right]}^{T}\;\;\to \;\;{\bar{\text{q}}}^{-1}=\frac{\left[-{\text{q}}^{T}\;q_{4}\right]}{{\left|\bar{\text{q}}\right|}^{2}}.$$

When the unit norm constraint Equation (6) is enforced, a quaternion is left with the three degrees of freedom consistent with the SO(3) dimensionality. The four-component unit quaternion has thus the lowest dimension of any globally non-singular orientation parameterization. However, the representation is redundant, since the quaternion −**q̄** represents the same rotation as **q̄**.

### 2.2. Kinematic Equations

These describe the relations between the temporal derivatives of an orientation representation such as the quaternion **q̄** and the angular velocity **ω**_ℬ_, namely the angular velocity of the ℬ-frame relative to the ℰ-frame, as it is measured by a tri-axial gyro fixed to the body:

(9) $$\frac{d}{\mathrm{dt}}\bar{\text{q}}=\frac{1}{2}\bar{\text{q}}⊗\;{\left[\omega_{ℬ}{}^{T}\;0\right]}^{T}=\frac{1}{2}\left[\begin{array}{cc} -\left[\omega_{ℬ}\times \right] & \omega_{ℬ}\\ -\omega_{ℬ}{}^{T} & 0 \end{array}\right]\bar{\text{q}}=\Omega \left(\omega_{ℬ}\right)\bar{\text{q}},$$

In the following the time argument will always be dropped to make the notation a little easier. **Ω**(**ω**_B_) is a 4 × 4 skew symmetric matrix. The kinematic Equation (9) do not involve nonlinear computationally expensive trigonometric functions and are not affected by the presence of singularity points, in contrast to the formulation using representations of orientation such as the Euler angles.

The discrete-time equivalent of Equation (9) is given by:

(10) $$\bar{\text{q}}(t_{k})=\Phi \bar{\text{q}}(t_{k-1}),$$

with:

(11) $$\Phi =\text{cos}\left(\left|{\text{u}}_{ℬ}\right|/2\right)\;{\text{I}}_{4\times 4}+\frac{\text{sin}\left(\left|{\text{u}}_{ℬ}\right|/2\right)}{\left|{\text{u}}_{ℬ}\right|/2}\Omega \left(\omega_{ℬ}(t_{k-1})\right).$$

If the angular velocity is assumed constant in the sampling interval *T_s_* = *t_k_* − *t*_*k*−1_, we have:

(12) $${\text{u}}_{ℬ}=\int_{t_{k-1}}^{t_{k}}\omega_{ℬ}(\tau )\;d\tau \approx \omega_{ℬ}(t_{k-1})\;T_{s}.$$

### 2.3. Observability Analysis

A system is observable when, for any possible sequence of state and control input vectors, the current state can be inferred by measuring the output variables. In other words, the output variables contain all the information needed to determine the behavior of the system. For continuous time-varying nonlinear systems the observability analysis, namely the analysis needed to verify whether a system is observable or not, requires the development of specific tools [25].

Consider a nonlinear system **Σ** described, in state space form, by a set of equations of the following form:

(13) $$\left\{ \begin{array}{l} \frac{d}{\mathrm{dt}}\text{x}=\text{f}\;\left(\text{x},\text{u}\right)={\text{f}}_{0}\;(\text{x})+\sum_{i=1}^{l}{\text{f}}_{i}\;(\text{x})u_{i}\\ \text{y}=\text{h}\left(\text{x}\right) \end{array}\right.$$

The time argument in **x**, **u**, and **y** is dropped. **x** = [*x*_1_, *x*_2_,…, *x_n_*]*^T^* ∈ **X** ⊂ ℝ*^n^*, **u** = [*u*_1_, *u*_2_,…, *u_l_*,]*^T^* ∈ ℝ*^l^*, **y** = [*y*_1_, *y*_2_,…, *y_m_*]*^T^* ∈ ℝ*^m^* are, respectively, the state vector, the control input vector and the measurement vector. The process function **f** is input-linear, *i.e.*, it can be decomposed into a sum of independent functions **f***_i_*, each one corresponding to a different component *u_i_* of the control input vector **u**. The process function **f** and the measurement function **h** are assumed smooth in their arguments, namely they possess continuous partial derivatives of any order.

A suitable tool for studying the observability properties of **Σ** is the observability rank condition based on the Lie derivatives of the measurement functions *h_k_*(**x**), *k* = 1,...,*m*. The zero-order Lie derivative of a scalar function is, by definition, the function itself, ℒ^0^ *h_k_*(**x**) = *h_k_*(**x**), *k* = 1,…,*m*. The first-order Lie derivative of the measurement function *h_k_*(**x**) with respect to the component **f***_i_* of the process function **f** is given by:

(14) $${ℒ}_{{\text{f}}_{i}}^{1}h_{k}\;(\text{x})=\nabla h_{k}\;(\text{x})\cdot {\text{f}}_{i}\;(\text{x})=\sum_{j=1}^{n}\frac{\partial h_{k}}{\partial x_{j}}(\text{x})\;f_{\mathrm{ij}}\;(\text{x}),\;\;i=0,...,l,$$

where ∇ denotes the gradient operator. Since the first-order Lie derivative is a scalar function itself, the second-order Lie derivative of *h_k_*(**x**) with respect to **f***_i_* is defined in a similar fashion to (14):

(15) $${ℒ}_{{\text{f}}_{i}}^{2}h_{k}\;(\text{x})=\nabla {ℒ}_{{\text{f}}_{i}}^{1}h_{k}\;(\text{x})\cdot {\text{f}}_{i}\;(\text{x}).$$

The definition of higher-order and mixed Lie derivatives with respect to different components **f***_i_*, **f***_j_* of the process function **f** follows the same route as above. For instance, the second-order Lie derivatives with respect to **f***_j_* and **f***_i_*, given their first-order derivatives with respect to **f***_i_*, are given by

(16) $${ℒ}_{{\text{f}}_{j}{\text{f}}_{i}}^{2}h_{k}\;(\text{x})=\nabla {ℒ}_{{\text{f}}_{i}}^{1}h_{k}\;(\text{x})\cdot {\text{f}}_{j}\;(\text{x}),\;i,j=0,...,l;\;\;\;k=1,...,m.$$

The observability matrix is constructed by stacking the gradients of the Lie derivatives:

(17) $$\text{O}(\text{x})=\left\{\nabla {ℒ}_{{\text{f}}_{i}\ldots {\text{f}}_{j}}^{s}\;h_{k}\;(\text{x})|i,j=0,...,l;\;k=1,...,m;\;s\in \mathbb{N}\right\}.$$

The number of columns of the observability matrix is equal to the dimension *n* of the state vector **x**, while the number of rows may grow unbounded with the order of the computed Lie derivatives. Since the input and measurement functions are smooth, the number of rows of **O** can be thus virtually infinite.

For nonlinear systems the observability property is related to the concept of indistinguishability of states with respect to the control inputs. Two states **x**_1_ and **x**_2_ are said indistinguishable when, for every admissible control input, the system produces the same outputs in a given interval of time when starting from either **x**_1_ or **x**_2_. The system **Σ** is weakly observable at the state **x**_0_ if for every open neighborhood *U* of **x**_0_, **x**_0_ can be distinguished from states in *U*(**x**_0_), or weakly observable if it is so for every **x** ∈ **X**. The system **Σ** is locally weakly observable at the state **x**_0_ if **x**_0_ can be distinguished from states in an open neighborhood *V*(**x**_0_) ⊂ *U*(**x**_0_) when admissible control inputs lead to paths within *U*(**x**_0_), or locally weakly observable if it is so for every **x** in **X**. A sufficient condition for the system **Σ** to be locally weakly observable at **x**_0_ is that **O**(**x**_0_) is full rank, namely the gradients of the Lie derivatives of the measurement functions computed at **x** = **x**_0_ must span the state space **X**. If it is so for every **x** ∈ **X** the system **Σ** is locally weakly observable. It should be noted that the full-rank condition of the observability matrix **O**(**x**) is only sufficient to say that the system is locally weakly observable. In the case that the observability matrix **O**(**x**) is rank-deficient, the system’s behavior has to be verified through computer simulations and experimental testing.

## 3. Method

### 3.1. Sensor Modeling

In response to the time-variant body angular velocity **ω**_body_, acceleration (constant gravity **g** and time-variant body acceleration **a**_body_), and local magnetic field (constant earth’s magnetic field **h** and any time-variant magnetic variation *^h^***b** acting nearby the sensor) the measured outputs of the gyro, the accelerometer and the magnetic sensor from an integrated IMU can be written as follows:

(18) $$\left\{ \begin{array}{l} \omega_{m}={}^{g}\;\text{K}\;\omega_{\text{body}}+{}^{g}\text{b}+{}^{g}\text{v}\\ {\text{a}}_{m}={}^{a}\text{K}{}_{ℰ}^{ℬ}\text{C}\left(-\text{g}+{\text{a}}_{\text{body}}\right)+{}^{a}\text{b}+{}^{a}\text{v}\\ {\text{h}}_{m}={}^{m}\text{K}{}_{ℰ}^{ℬ}\text{C}\left(\text{h}+{}^{h}\text{b}\right)+{}^{m}\text{b}+{}^{m}\text{v}, \end{array}\right.$$

*^g^***K**, *^a^***K**, *^m^***K** are the matrices of the scale factors (ideally, they are equal to **I**_3×3_); *^g^***b**, *^a^***b**, *^m^***b** are the bias vectors (ideally, they are null); *^g^***v**, *^a^***v**, *^m^***v** are assumed independent white Gaussian measurement noises, with null mean and covariance matrix $\Sigma_{g}=\sigma_{g}^{2}{\text{I}}_{3\times 3}$, $\Sigma_{a}=\sigma_{a}^{2}{\text{I}}_{3\times 3}$ and $\Sigma_{m}=\sigma_{m}^{2}{\text{I}}_{3\times 3}$. Equation (18) is a simplified model that does not account for additional error sources, such as cross-axis sensitivity, gyro *g*-sensitivity, nonlinearity, hysteresis and misalignment [26]. In the context of MEMS sensors, the component in the gyro output due to the Earth’s rotation can be neglected as compared to sensor errors, and therefore it does not appear in Equation (18).

The scale factor and bias of inertial and magnetic sensors are functions of environmental conditions, in particular ambient temperature. Across the thermal variations typically encountered in practice, thermal effects on accelerometers are of relatively lower quantitative relevance than on gyros, and they are usually negligible on magnetic sensors. Moreover, scale factor drifts of inertial and magnetic sensors usually affect the accuracy of the measurement process to a much lesser extent than the bias drifts of these sensors, in particular gyros. Scale factor and bias errors of accelerometers can be compensated on time scales up to few hours, using the procedure described in [26]; the procedure described in [27] can be used to calibrate scale factors and hard iron offsets *^m^***b**, on similar time scales.

A more serious problem concerns the bias errors of gyros and how they develop over time. This problem is well received in the literature, where orientation estimators are oftentimes developed with built-in devices for gyro-bias compensation. A random-walk vector random process with statistically independent components is widely used for carrying the compensation task in a Kalman filtering framework [28]:

(19) $$\frac{d}{\mathrm{dt}}{}^{g}\text{b}={\text{w}}_{g},$$

where **w***_g_* is white Gaussian noise, with null mean and covariance matrix ${}^{b}\Sigma_{g}={}^{b}\sigma_{g}^{2}\;{\text{I}}_{3\times 3}$. We consider the implementation of these devices essential for a proper operation of the filter, especially when the opportunity to perform on-line bias capture are precluded by the nature of the tracked motions [29].

For slowly moving bodies in magnetically clean environments, gyro-free orientation determination systems have been developed with mixed success [3,11]. In these systems a further simplification is made in the sensor model Equation (18), where the only acceleration measured by the accelerometer is gravity. Henceforth, we make the simplifying assumption that **a**_body_ ≈ **0**.

### 3.2. Modeling the Magnetic Variation

A magnetic sensor measures the Earth’s magnetic field plus any other magnetic field superimposed to it. For the purpose of orientation determination, an accurately known homogeneous magnetic field in the environment surrounding the tracked body is needed as the horizontal reference for heading estimation. Especially indoors, magnetic homogeneity is difficult to achieve, due to construction iron in floors, walls and ceilings, or to various equipment. The magnetic distortions occur in both the horizontal and vertical plane [6,7]. Suppose that, albeit not homogeneous, the magnetic field existing in a given region of space is at least time-invariant. In this case, an interesting possibility to deal with the problem of magnetic distortions would consist, in principle, of calibrating and mapping the measurement volume. Besides being complicated and time-consuming, this approach is also in contrast with the development of ambulatory sensor systems, whose prior knowledge of existence and location of disturbances cannot be taken for granted: for instance, current IMUs do not produce position information, which makes critical using the magnetic maps of the measurement volume.

IMUs are exposed to magnetic fields that can rapidly change in direction and magnitude, when they move relative to their surroundings in a magnetically non-homogeneous environment. These magnetic distortions, generically denoted with the term magnetic variation *^h^***b**, can be expressed in the earth-fixed frame. In order to prevent heading drift, the orientation estimator has to be developed with a built-in device for compensation of magnetic variations. A first-order Gauss-Markov vector random process with statistically independent components is chosen to model the magnetic variation:

(20) $$\frac{d}{\mathrm{dt}}{}^{h}\text{b}=-\alpha \;{}^{h}\text{b}+{\text{w}}_{h},$$

where *α* is a positive constant, and **w***_h_* is white Gaussian noise, with null mean and covariance matrix ${}^{b}\Sigma_{h}={}^{b}\sigma_{h}^{2}\;{\text{I}}_{3\times 3}$. This model is the same considered in [17], and generalizes the random-walk model adopted in our previous research [2]. The random-walk model is obtained by Equation (20) by taking *α* = 0.

### 3.3. Filter Implementation

The main difficulty of using quaternion-based state vector components in an EKF lies in the formulation of the filter equations. For a review of these equations with a generic state-space model see [30]. The origin of the problem lies in the lack of independence of the four components of a quaternion, since they are related by the unit-norm constraint. Constraints imposed on the estimated state variables cannot be preserved by EKFs in their standard development [19]. A popular method to preserve the quaternion unit-norm property is to normalize the *a posteriori* estimate after the measurement update stage (“brute-force” approach). Even though it is neither elegant nor optimal [31], the “brute-force” approach is proven to work generally well [32]. The EKF developed in this paper enforces the unit-norm constraint by the “brute-force” approach [2].

Since the angular velocity is measured from a body-fixed tri-axial gyro, the kinematic equations of a rigid body can be used to obtain the orientation state [19]. Gyro data are treated as external inputs to the filter rather than as measurements, and gyro measurement noise and bias enter the filter as process noise rather than as measurement noise. An advantage of this choice is the reduction in the dimension of the state vector, which may lead to minimal-order, computationally efficient filter implementations.

An important feature of EKFs is the possibility they offer to estimate unknowns by state vector augmentation techniques. In this paper, we concentrate on the self-compensation of gyro bias and magnetic variation, both accounted for in the filter as additional state vector components.

The continuous-time system model combines (9)-(19)-(20) together:

(21) $$\left\{ \begin{array}{l} \frac{d}{\mathrm{dt}}\bar{\text{q}}=\Omega (\omega )\bar{\text{q}}\\ \frac{d}{\mathrm{dt}}{}^{h}\text{b}=-\alpha \;{}^{h}\text{b}+{\text{w}}_{h}\\ \frac{d}{\mathrm{dt}}{}^{g}\text{b}={\text{w}}_{g}. \end{array}\right.$$

The state vector in Equation (21) is $\text{x}={[\begin{array}{lll} {\bar{\text{q}}}^{T} & {}^{h}{\text{b}}^{T} & {}^{g}{\text{b}}^{T}] \end{array}}^{T}$. The equations for propagating the state estimates in the model are obtained by applying the expectation operator to Equation (21). By rearranging the equations in a format suitable for computing the Lie derivatives [23], we obtain:

(22) $$\left[\begin{array}{c} \frac{d}{\mathrm{dt}}\hat{\bar{\text{q}}}\\ \frac{d}{\mathrm{dt}}{\hat{\text{b}}}_{h}\\ \frac{d}{\mathrm{dt}}{\hat{\text{b}}}_{g} \end{array}\right]=\underset{{\text{f}}_{0}}{\underset{︸}{\left[\begin{array}{c} -\frac{1}{2}\Xi \left(\hat{\bar{\text{q}}}\right){\hat{\text{b}}}_{g}\\ -\alpha \;{\hat{\text{b}}}_{h}\\ 0_{3\times 1} \end{array}\right]}}+\underset{{\text{f}}_{1}}{\underset{︸}{\left[\begin{array}{c} \frac{1}{2}\Xi \left(\hat{\bar{\text{q}}}\right)\\ 0_{3\times 1}\\ 0_{3\times 1} \end{array}\right]}}\omega_{m},$$

where the angular velocity **ω̂** = **ω***_m_* − *^g^***b̂** is treated as a control input, and the matrix **Ξ**(**q̄**) is given by:

(23) $$\Xi \left(\bar{\text{q}}\right)=\left[\begin{array}{c} q_{4}{\text{I}}_{3\times 3}+\left[\text{q}\times \right]\\ -{\text{q}}^{T} \end{array}\right]=\left[\begin{array}{ccc} q_{4} & -q_{3} & q_{2}\\ q_{3} & q_{4} & -q_{1}\\ -q_{2} & q_{1} & q_{4}\\ -q_{1} & -q_{2} & -q_{3} \end{array}\right]=\left[\begin{array}{ccc} {\bar{\text{s}}}_{1} & {\bar{\text{s}}}_{2} & {\bar{\text{s}}}_{3} \end{array}\right]$$

In the following we drop the caret, which denotes the expectation of a random variable, to avoid unnecessary cluttering in the notation. The discrete-time model allows employing the sampled measurements of the IMU for state propagation:

(24) $$\overset{\text{x}(k)}{\overset{︷}{\begin{array}{c} \bar{\text{q}}(k)\\ \left[\begin{array}{c} {}^{h}\;\text{b}(k)\\ {}^{g}\;\text{b}(k) \end{array}\right] \end{array}}}=\overset{\text{f}(k-1)}{\overset{︷}{\begin{array}{c} \Phi (k-1)\\ \left[\begin{array}{cc} \text{exp}(-\alpha T_{s}){\text{I}}_{3\times 3} & 0_{3\times 3}\\ 0_{3\times 3} & {\text{I}}_{3\times 3} \end{array}\right] \end{array}}}\overset{\text{x}(k-1)}{\overset{︷}{\begin{array}{c} \bar{\text{q}}(k-1)\\ \left[\begin{array}{c} {}^{h}\text{b}(k-1)\\ {}^{g}\text{b}(k-1) \end{array}\right] \end{array}}}\overset{\text{w}(k-1)}{\overset{︷}{\begin{array}{c} {}^{q}\text{w}(k-1)\\ \left[\begin{array}{c} {}^{h}\;\text{w}(k-1)\\ {}^{g}\text{w}(k-1) \end{array}\right] \end{array}}}$$

where:

(25) $$\begin{array}{l} \Phi (k-1)=\text{cos}\left(\left|\tilde{\omega }(k-1)\right|T_{s}/2\right){\text{I}}_{4\times 4}+\frac{\text{sin}\left(\left|\tilde{\omega }(k-1)\right|T_{s}/2\right)}{\left|\tilde{\omega }(k-1)\right|T_{s}/2}\Omega \left(\tilde{\omega }(k-1)\right)\\ \tilde{\omega }(k-1)=\omega_{m}(k-1){-}^{g}\text{b}(k-1). \end{array}$$

The process noise component *^q^***w**(*k* − 1) describes how the gyro measurement noise enters the state model through a quaternion-dependent linear transformation, as follows:

(26) $${}^{q}\text{w}(k-1)=-\frac{T_{s}}{2}\Xi \;\left(\bar{\text{q}}(k-1)\right){}^{g}\text{v}(k-1)$$

The process noise components *^q^***w**(*k* − 1), *^h^***w**(*k* − 1), *^g^***w**(*k* − 1) are assumed uncorrelated; hence, the process noise covariance matrix **Q**(*k* − 1) is shown to have the block-diagonal structure:

(27) $$\text{Q}(k-1)=\left[\begin{array}{ccc} \sigma_{g}^{2}{\left(T_{s}/2\right)}^{2}\left(\text{trace}({\text{M}}_{o})\;{\text{I}}_{4\times 4}-{\text{M}}_{o}\right) & 0_{3\times 3} & 0_{3\times 3}\\ 0_{3\times 3} & \sigma_{h}^{2}\frac{1-\text{exp}\left(-2\alpha \;T_{s}\right)}{2\alpha }{\text{I}}_{3\times 3} & 0_{3\times 3}\\ 0_{3\times 3} & 0_{3\times 3} & \left({}^{b}\sigma_{g}^{2}T_{s}\right){\text{I}}_{3\times 3} \end{array}\right]$$

where:

(28) $${\text{M}}_{o}=\bar{\text{q}}(k-1)\bar{\text{q}}{\left(k-1\right)}^{T}+{\text{P}}^{q}(k-1)$$

**q̄**(*k* − 1) and **P***^q^*(*k* − 1) denote, respectively, the expectation and the covariance matrix of the quaternion component of the state vector [32].

Equation (18) is used to model the sensor measurements. Each reference vector component needed for vector matching is given a specific equation:

(29) $$\left[\begin{array}{c} {\text{a}}_{m}(k)\\ {\text{h}}_{m}(k) \end{array}\right]=\left[\begin{array}{cc} {}_{ℰ}^{ℬ}\text{C}\left(\bar{\text{q}}(k)\right) & 0_{3\times 3}\\ 0_{3\times 3} & {}_{ℰ}^{ℬ}\text{C}\left(\bar{\text{q}}(k)\right) \end{array}\right]\left[\begin{array}{c} \text{g}\\ \text{h}+{}^{h}\text{b}(k) \end{array}\right]+\left[\begin{array}{c} {}^{a}\text{v}(k)\\ {}^{m}\text{v}(k) \end{array}\right]$$

as done in [2]. The measurement equations are nonlinear, which forces to compute their Jacobian matrices when carrying out the linearization process implied by the EKF. They will be shown in the next Section, in connection with the problem of computing the Lie derivatives of the nonlinear system of Equation (22). The measurement noise covariance matrix can be expressed directly in terms of the statistics of the measurement noise affecting each sensor:

(30) $$\text{R}(k)={\left[\begin{array}{cc} \sigma_{a}^{2}{\text{I}}_{3\times 3} & 0\\ 0 & \sigma_{m}^{2}{\text{I}}_{3\times 3} \end{array}\right]}_{.}$$

No vector selection scheme is introduced in the filter developed in this paper [2].

The initialization procedure is carried out as follows: the processing of gyro data does not include the procedure of bias capture; an initialization error is thus introduced by setting the gyro bias component of the state vector to zero. The magnetic field is not taken from a magnetic field model, but is rather computed as follows. The aiding sensor measurements are averaged during the initial rest period (averaging time: 1s). Inclination is estimated by processing the acceleration average vector. The magnetic average vector is then projected in the horizontal plane using the estimated inclination, which allows estimating **h** in the earth-fixed frame. Finally, we apply the TRIAD method to the acceleration and magnetic average vectors, using the gravity vector **g** and the estimated value of **h** as reference vectors [3,33]. The initial quaternion and its covariance matrix are then computed.

### 3.4. Filter Observability

The observability test consists of demonstrating that the state space of the system with input-linear process function described by Equation (22) is spanned by the gradients of the Lie derivatives of the following measurement functions:

(31) $$\begin{array}{l} \varphi_{1}(\text{x})={}_{ℰ}^{ℬ}\text{C}\left(\bar{\text{q}}\right)\;\left(-\text{g}\right)\\ \varphi_{2}(\text{x})={}_{ℰ}^{ℬ}\text{C}\left(\bar{\text{q}}\right)\;\left(\text{h}+{}^{h}\text{b}\right)\\ \varphi_{3}(\text{x})={\bar{\text{q}}}^{T}\bar{\text{q}}-1 \end{array}$$

The measurement function *φ*_3_(**x**) is introduced to account for the quaternion normalization constraint [23]. For its relevance in performing the observability analysis, we introduce, for a generic vector **p**, the function:

(32) $$\text{ψ}\left(\bar{\text{q}},\;\text{p}\right)=\frac{\partial }{\partial \bar{\text{q}}}{}_{ℰ}^{ℬ}\text{C}\left(\bar{\text{q}}\right)\;\text{p}.$$

Using Equation (2) we obtain:

(33) $$\text{ψ}\left(\bar{\text{q}},\;\text{p}\right)=\left[2q_{4}\left[\text{p}\times \right]+2\left(\left[\text{p}\times \right]\left[\text{q}\times \right]-2\left[\text{q}\times \right]\left[\text{p}\times \right]\right)\;-2\left[\text{q}\times \right]\text{p}\right]$$

Since the zero-order Lie derivatives are the measurement functions themselves, the gradients of the zero-order Lie derivatives are the Jacobian matrices of the measurement functions:

(34) $$\begin{array}{l} \nabla {ℒ}^{0}\varphi_{1}(\text{x})=\left[\begin{array}{ccc} \psi \left(\bar{\text{q}},-\text{g}\right) & 0_{3\times 3} & 0_{3\times 3} \end{array}\right]\\ \nabla {ℒ}^{0}\varphi_{2}(\text{x})=\left[\begin{array}{ccc} \psi \left(\bar{\text{q}},\text{h}+{}^{h}\text{b}\right) & {}_{ℰ}^{ℬ}\text{C}\left(\bar{\text{q}}\right) & 0_{3\times 3} \end{array}\right]\\ \nabla {ℒ}^{0}\varphi_{3}(\text{x})=\left[\begin{array}{ccc} 2{\bar{\text{q}}}^{T} & 0_{1\times 3} & 0_{1\times 3} \end{array}\right] \end{array}$$

The first-order Lie derivatives of **φ**_1_ and **φ**_2_ (with respect to **f**_0_) can be computed as:

(35) $$\begin{array}{l} {ℒ}_{{\text{f}}_{0}}^{1}\varphi_{1}(\text{x})=\nabla {ℒ}^{0}\varphi_{1}(\text{x})\cdot {\text{f}}_{0}=-\frac{1}{2}\psi \left(\bar{\text{q}},-\text{g}\right)\;\Xi \left(\bar{\text{q}}\right){\text{b}}_{g}\\ {ℒ}_{{\text{f}}_{0}}^{1}\varphi_{2}(\text{x})=\nabla {ℒ}^{0}\varphi_{2}(\text{x})\cdot {\text{f}}_{0}=-\frac{1}{2}\psi \left(\bar{\text{q}},\text{h}+{}^{h}\text{b}\right)\;\Xi \left(\bar{\text{q}}\right)\;{\text{b}}_{g}+{}_{ℰ}^{ℬ}\text{C}\left(\bar{\text{q}}\right)\left(-\alpha \;{}^{h}\text{b}\right) \end{array}$$

The gradients of the first-order Lie derivatives of **φ**_1_ and **φ**_2_ (with respect to **f**_0_) are then taken:

(36) $$\begin{array}{l} \nabla {ℒ}_{{\text{f}}_{0}}^{1}\varphi_{1}(\text{x})=\left[\begin{array}{ccc} {\text{X}}_{1} & 0_{3\times 3} & -\frac{1}{2}\psi \left(\bar{\text{q}},-\text{g}\right)\;\Xi \left(\bar{\text{q}}\right) \end{array}\right]\\ \nabla {ℒ}_{{\text{f}}_{0}}^{1}\varphi_{2}(\text{x})=\left[\begin{array}{ccc} {\text{X}}_{2} & {\text{X}}_{2} & -\frac{1}{2}\psi \left(\bar{\text{q}},\text{h}+{}^{h}\text{b}\right)\;\Xi \left(\bar{\text{q}}\right) \end{array}\right] \end{array}$$

The next Lie derivative of interest is the first-order Lie derivative of **φ**_2_ with respect to **f**_1_:

(37) $${ℒ}_{\;{\text{f}}_{1}}^{1}\varphi_{2}(\text{x})=\nabla {ℒ}^{0}\varphi_{2}(\text{x})\cdot {\text{f}}_{1}=\frac{1}{2}\psi \left(\bar{\text{q}},\text{h}+{}^{h}\text{b}\right)\;\Xi \left(\bar{\text{q}}\right)$$

The gradient of the first-order Lie derivative of **φ**_2_ with respect to **f**_1_ is obtained by expanding the Lie derivatives as column vectors. These column vectors are stacked together as follows:

(38) $$\begin{array}{l} \nabla {ℒ}_{{\text{f}}_{1}}^{1}\varphi_{2}(\text{x})=\left[\begin{array}{ccc} \Gamma & ϒ & 0_{9\times 3} \end{array}\right]\\ \Gamma =\left[\begin{array}{c} \Gamma_{1}\\ \Gamma_{2}\\ \Gamma_{3} \end{array}\right]=\frac{1}{2}\left[\begin{array}{c} \frac{\partial }{\partial \bar{\text{q}}}\left(\psi \left(\bar{\text{q}},\text{h}+{}^{h}\text{b}\right)\;\Xi \left(\bar{\text{q}}\right){\text{e}}_{1}\right)\\ \frac{\partial }{\partial \bar{\text{q}}}\left(\psi \left(\bar{\text{q}},\text{h}+{}^{h}\text{b}\right)\;\Xi \left(\bar{\text{q}}\right){\text{e}}_{2}\right)\\ \frac{\partial }{\partial \bar{\text{q}}}\left(\psi \left(\bar{\text{q}},\text{h}+{}^{h}\text{b}\right)\;\Xi \left(\bar{\text{q}}\right){\text{e}}_{3}\right) \end{array}\right]\;\;ϒ=\left[\begin{array}{c} {ϒ}_{1}\\ {ϒ}_{2}\\ {ϒ}_{3} \end{array}\right]=\frac{1}{2}\left[\begin{array}{c} \frac{\partial }{\partial {}^{h}\text{b}}\left(\psi \left(\bar{\text{q}},{}^{h}\text{b}\right)\;\Xi \left(\bar{\text{q}}\right){\text{e}}_{1}\right)\\ \frac{\partial }{\partial {}^{h}\text{b}}\left(\psi \left(\bar{\text{q}},{}^{h}\text{b}\right)\;\Xi \left(\bar{\text{q}}\right){\text{e}}_{2}\right)\\ \frac{\partial }{\partial {}^{h}\text{b}}\left(\psi \left(\bar{\text{q}},{}^{h}\text{b}\right)\;\Xi \left(\bar{\text{q}}\right){\text{e}}_{3}\right) \end{array}\right] \end{array}$$

where **e**_1_ = [1 0 0]*^T^*, **e**_2_ = [0 1 0]*^T^*, **e**_3_ = [0 0 1]*^T^*.

Finally, we obtain the 22 × 10 observability matrix **O**(**x**):

(39) $$\text{O}(\text{x})=\left[\begin{array}{c} \nabla {ℒ}_{0}\varphi_{1}(\text{x})\\ \nabla {ℒ}_{0}\varphi_{2}(\text{x})\\ \nabla {ℒ}_{f_{0}}\varphi_{1}(\text{x})\\ \nabla {ℒ}_{f_{0}}\varphi_{2}(\text{x})\\ \nabla {ℒ}_{f_{1}}\varphi_{2}(\text{x})\\ \nabla {ℒ}_{0}\varphi_{3}(\text{x}) \end{array}\right]=\left[\begin{array}{cc} {\text{C}}_{6\times 7} & 0_{6\times 3}\\ {\text{D}}_{6\times 7} & {\text{A}}_{6\times 3}\\ {\text{B}}_{10\times 7} & 0_{10\times 3} \end{array}\right]$$

where the following matrices are introduced:

(40) $$\begin{array}{l} \text{A}=\left[\begin{array}{c} {\text{A}}_{11}\\ {\text{A}}_{21} \end{array}\right]=\left[\begin{array}{c} -\frac{1}{2}\psi \left(\bar{\text{q}},-\text{g}\right)\;\Xi \left(\bar{\text{q}}\right)\\ -\frac{1}{2}\psi \left(\bar{\text{q}},\text{h}+{}^{h}\text{b}\right)\;\Xi \left(\bar{\text{q}}\right) \end{array}\right]\;\text{B}=\left[\begin{array}{cc} \Gamma & ϒ\\ 2{\bar{\text{q}}}^{T} & 0_{1\times 3} \end{array}\right]\;\;\\ \text{C}=\left[\begin{array}{cc} \psi \left(\bar{\text{q}},-\text{g}\right) & 0_{3\times 3}\\ \psi \left(\bar{\text{q}},\text{h}+{}^{h}\text{b}\right) & {}_{ℰ}^{ℬ}\text{C}\left(\bar{\text{q}}\right) \end{array}\right]\;\text{D}=\left[\begin{array}{cc} {\text{X}}_{1} & 0_{3\times 3}\\ {\text{X}}_{2} & {\text{X}}_{3} \end{array}\right] \end{array}$$

The salient steps of the observability proof are reported in Appendix A, where the matrices **X**_1_, **X**_2_, **X**_3_, and hence the matrix **D**, do not need to be computed explicitly. In short, the system of Equation (22) is *certainly* locally weakly observable when: (a) the magnetic field has components in the horizontal plane, which is necessary to have the horizontal reference for heading estimation; (b) at least one degree of rotational freedom is excited. Remind that the magnetic dip angle, also called magnetic inclination, is the angle the Earth’s magnetic field makes with the surface of the Earth; the observability condition (a) fails when the dip angle is ±90° (e.g., close to the Poles). Running the estimation algorithm during periods when the observability is not guaranteed—when the magnetic dip angle is close to ±90° or the IMU is in quasi-static conditions—is likely to degrade seriously the state estimation accuracy. This fact has to be verified by assessing the system’s behavior by computer simulations and experimental testing.

### 3.5. Filter Assessment

The performance metrics are based on computing the error quaternion $\Delta \bar{\text{q}}={\bar{\text{q}}}_{t}^{-1}⊗\bar{\text{q}}$, where **q̄**_t_ and **q̄** are the true and the estimated quaternion, respectively. In the present context the term *true* implies the use of synthetic or experimental motion data to carry out the test procedure. An obvious advantage of working with synthetic signals is that the errors incurred in estimating the state vector components can be compared with the bounds that are predicted by the error covariance matrix produced by the EKF. This is a useful feature to assess the filter convergence and to diagnose a number of potential problems arising in its numerical implementation. Experimental motion data can be captured, e.g., from an optoelectronic motion tracker, so as to define the ground truth reference for filter assessment.

The error quaternion represents the rotation that brings the estimated body frame onto the true body frame. Its scalar component can be used to derive the orientation error Δ*θ*, according to the equation Δθ = 2arccos(Δ*q*_4_) The performance metrics are expressed in terms of the root-mean-square-value of the orientation error (RMSE*_q_*), averaged over the number of either the Monte Carlo simulation runs or the experimental trials available. Alternatively, a set of estimated and reference Euler angles (roll, pitch, yaw or heading) can be computed from **q̄**_t_ and **q̄** using standard conversion formulas, and the filter performance can be summarized by presenting the RMSEs of the Euler angles, again averaged over the number of either the Monte Carlo simulation runs or the experimental trials available. Although the calculations for orientation are not performed using Euler angles, the results can be presented in this way for better interpretation.

### 3.6. Computer Simulations

The body-fixed frame has a fixed origin, and it is aligned with the Earth-fixed frame at the initial time of each simulation trial. Our choice of the earth-fixed frame follows the North-East-Down (NED) convention: the gravity vector is **g** = [0 0 *g*]^T^ (*g* = 9.81 m/s^2^), and the earth magnetic field is **h** = [*h_x_* 0 *h_z_*]^T^ (*h_x_* = 0.26 Gauss; *h_z_* = 0.37 Gauss). The simulated field strength is approximately 0.45 Gauss, with a magnetic dip angle *δ* ≈ 55°, situations typical of our latitudes. A magnetically perturbed environment is simulated by adding **h** with the output of a routine that generates samples from a Gauss-Markov vector random process with statistically independent components.

The angular velocity time functions are given as input to the simulation program. The true quaternion time functions are obtained by time integrating the kinematic Equation (9) at the sampling frequency of *f_s_* = 4 kHz, which is high enough to make integration errors negligible; the quaternion time functions are then time-decimated down to *f_s_* = 100 Hz (the sampling frequency used in many popular orientation trackers). Two distinct conditions are tested: static conditions, in which case the angular velocity is null, and then the IMU does not change the initial orientation; dynamic conditions, *i.e.*, following an initial period of rest, a pure sinusoidal rotation around the vertical axis is simulated (amplitude: 100°/s; frequency: 1 Hz). In either case simulation trials last 10 min.

The magnetic field and the gravity vector can be expressed in the body-frame using the computed quaternions. White Gaussian noise is then injected into the body-referenced sensor time functions to simulate the effect of specified amounts of measurement noise: *σ_a_* = 1 − 5 m*g; σ_h_* = 1 mGauss. In dynamic conditions the value of *σ_a_* is chosen relatively high as compared with values measured during on-bench calibration tests of MEMS accelerometers, which are less than 1 m*g*; this is to account for the minute motions that affect on-body IMUs, even when the body is quasi-static. No scale factor or bias errors are considered for the IMU sensors, with the exception of the gyro: the gyro time functions are corrupted by addition of a Gaussian white measurement noise component with standard deviation *σ_g_* = 0.4°/s and a fixed offset (e.g., **b***_g_* = [1 − 0.5 0.75]*^T^* °/s).

The different treatment of IMU sensors is motivated by the weight an uncompensated gyro bias has on the error budget of an inertial orientation sensor and consequently by our desire to always enable the compensation of gyro bias in our filter. The following two EKF implementations are considered: EKF-M_A_ and EKF-M_B_, which differ in enabling the compensation of magnetic disturbances or not; in order to disable the compensation of magnetic disturbances, *^b^σ_h_* = 0 and *α* = 0 are plugged in Equation (27). The two methods are tested in static and dynamic conditions, when either the magnetic variation is markovian, namely it follows the Gauss-Markov model of Equation (20) (magnetically perturbed environment, MPE), or when the magnetic variation is null (magnetically clean environment, MCE). For each combination of method and condition, *N* = 10 Monte Carlo runs are performed, and the values of orientation RMSE*_q_* are reported (mean ± standard deviation, SD). Another set of simulations is carried for different values of the magnetic inclination, from 55° to 90° (dynamic conditions—method M_A_). The aim is to verify the filter behavior in difficult observability conditions. The filter parameter setting is given in Table 1.

### 3.7. Experimental Validation

The MTx orientation tracker by Xsens Technologies B.V. (Enschede, The Netherlands) is used for carrying out the experimental validation. The raw sensor data are delivered through a USB interface to the host computer at a rate of 100 Hz, together with the Euler angle time functions estimated by the proprietary EKF, henceforth called the Xsens-EKF. The IMU sensors are calibrated before starting the experimental session as described in [26,27]; by contrast, no bias capture is considered for the gyro. The initial orientation of the sensor frame relative to the marker frame is found by taking data during the rest period when the IMU is still.

The tests for validation in static and dynamic conditions are carried out within one of our lab rooms. The static test, whose duration is 10 min, consists of leaving the IMU still on a table that is placed far from current wires, computer appliances, and ferromagnetic materials. A magnetic disturbance is induced at the time when a cell phone is placed close to the IMU; the cell phone is left in place for a while, then it is removed, so as to recover the initial magnetic field. The test for validation in dynamic conditions is performed as follows. The IMU is fastened to a 50 cm 50 cm wooden plate using double-side adhesive tape. The plate is raised by hand slightly over the table and then it is freely moved around; toward the end of the trial, which lasts about 137 s, the plate is replaced approximately in the same pose. The working volume visited by the plate during the motion trial is 100 cm × 50 cm × 60 cm. The plate orientation is recorded using a six-camera Vicon optical motion capturing system with a sampling rate of *f_s_* = 100 Hz. The IMU and Vicon data streams are electronically time-synchronized. The Vicon system measures the position of four reflective markers (diameter: 14 mm) arranged at the corners of the plate to form a square with side 45 cm. The accuracy of the marker frame is determined by analyzing the relative motion of two markers: the RMS of the Vicon orientation error *Δθ* is assumed in the same order as the RMS distance variation divided by the distance between two markers (*Δθ* < 0.5°). The RMSEs of the Euler angles and the RMSE*q* of the orientation are presented using the Euler angles time functions computed by the Vicon system as the truth reference. The filter parameter setting we choose for the experimental validation is reported in Table 2. The orientation sensor delivers the data from the magnetic sensor in arbitrary units.

## 4. Results

### 4.1. Computer Simulations

**Figure 1.**
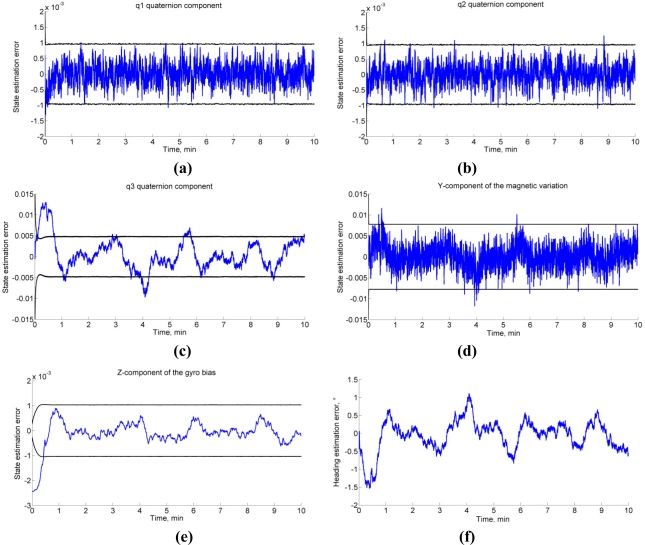
**(a)–(e)** State errors from the EKF. **(f)** Heading estimation error. **(a)–(c)** Quaternion components q_1_, q_2_, q_3_. **(d)** Magnetic variation *Y*-component. **(e)** Gyro bias *Z*-component. The black lines show the 3 standard deviation bounds estimated by the filter.

**Figure 2.**
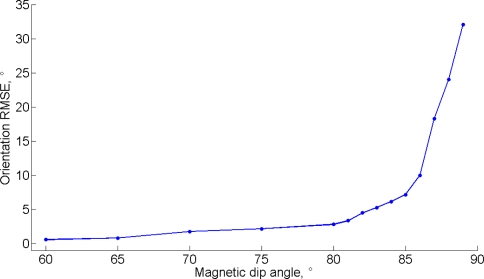
Orientation RMSE *vs.* magnetic dip angle.

The Monte Carlo results are reported in Table 3. The EKF-M_A_ performs better than the EKF-M_B_ in a magnetically markovian environment, according to a paired-sample *t*-test (statistically significant difference, SSD at *p* < 0.001 and *p* < 0.01, in dynamic and static conditions). In the case of null magnetic variations, the EKF-M_A_ performs as the EKF-M_B_. Figure 1 depicts the typical behavior in dynamic conditions. As for the second condition of observability, the EKF-M_A_ is tested in the same conditions leading to the data in Figure 1, for different values of the magnetic dip angle, Figure 2.

### 4.2. Experimental Validation: Static Test

**Figure 3.**
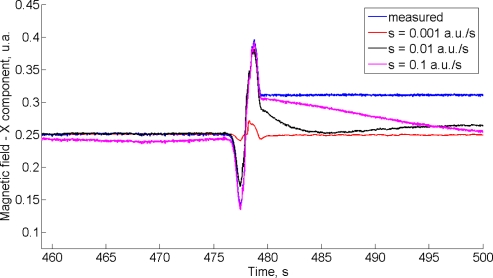
The time function of the *X*-component of the magnetic field, estimated by the EKF-M_A_. In blue the time function of the measured *X*-component of the magnetic field (see text).

In Figure 3 we plot, in blue, the component of the magnetic field along the *X*-direction (earth-fixed frame), as is measured by the magnetic sensor. Since the test environment is almost magnetically unperturbed before placing the cell phone nearby the IMU at time *t*_1_ = 476 s, the measured magnetic field is the true magnetic field existing in the room with very good approximation. The EKF-M_A_ is tested using three different values of the process noise standard deviation *^b^σ_h_*.

**Figure 4.**
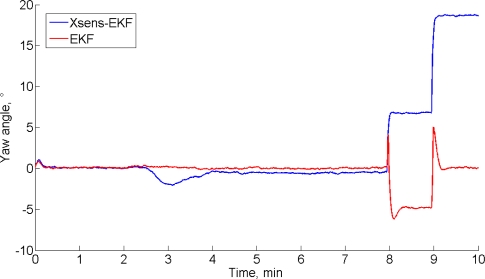
Heading angle time function estimated by the EKF-M_A_ and by the Xsens-EKF (*^b^σ_h_* = 0.01 a.u./s)

All the other parameters being the same as in Table 2, these values are: *^b^σ_h_* = 0.001 a.u./s (red); *^b^σ_h_* = 0.01 a.u./s (black); *^b^σ_h_* = 0.1 a.u./s (magenta). The RMS value of the difference between estimated and measured *X*-components of the magnetic field up to time *t*_1_ increases from 15 × 10^−5^ a.u. (*^b^σ_h_* = 0.001 a.u./s) to 8.4 × 10^−3^ a.u. (*^b^σ_h_* = 0.1 a.u./s). The magnetic disturbance from *t*_1_ to when the cell phone comes to a stop at time *t*_2_ = 480 s is best tracked with higher values of *^b^σ_h_*. However, when the magnetic field is not time variant anymore from time *t*_2_ on, the filter behavior is unsatisfactory. The estimated heading angle is reported in Figure 4.

### 4.3. Experimental Validation: Dynamic Test

**Figure 5.**
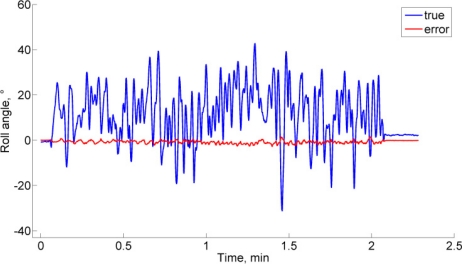

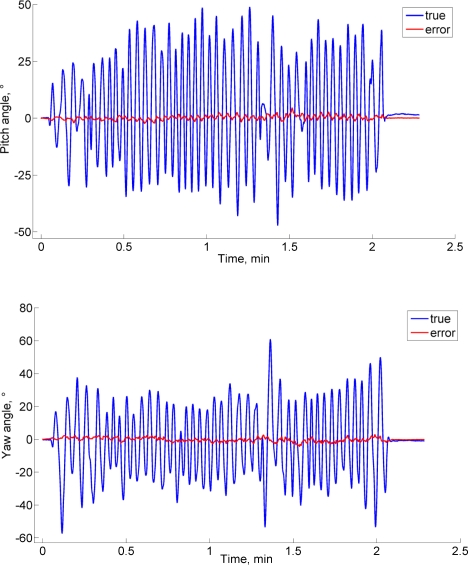
Euler angles time functions (truth reference and EKF-M_A_ estimation errors).

The norm of the acceleration magnitude is 1 *g* ± 60 m*g* throughout the trial, and the coefficient of variation of the norm of the measured magnetic field is about 1%. The RMSE of the Euler angles are reported in Table 4 for the EKF-M_A_ and the EKF-M_B_. As a matter of comparison, we also report the errors statistics in three other cases: (a) when only the gyro angular velocities are used and the aiding sensors are disabled; (b) when only the accelerometer and magnetic sensor data are involved in determining the orientation using the TRIAD method; (c) when the orientation is estimated by the Xsens-EKF. The Euler angle time functions estimated by the Vicon system are reported with superimposed the errors incurred by the EKF-M_A_ in Figure 5.

## 4. Discussion and Concluding Remarks

The conditions for the observability of the KF-based algorithm for IMU orientation tracking are based on the rank-condition of the observability matrix Equation (39). These conditions require that the local magnetic field, which may include magnetic variations superimposed to the earth magnetic field, and the gravity vector are not collinear; moreover, at least one rotational degree of freedom would excite the system. The computer simulations clearly show the relevance of the first condition. Indeed, when the magnetic dip angle becomes too large, the heading estimation accuracy undergoes an impressive degradation. Conversely, the computer simulations, and experimental testing as well, demonstrate that the condition concerning the IMU motion is not critical. In static conditions, the filter is stable and its performances are similar to those achieved in dynamic conditions. In general, we observe some problems of filter consistency. In particular, difficulties exist in picking up the third component of the quaternion and the horizontal magnetic states, as shown in Figure 1(c–d). The errors in these estimated state vector components turn out to be highly correlated in time, which is not reflected in the expression of the computed error state covariance matrix; moreover, while the compensation of gyro bias is generally fast, some possible problems exist for its *Z*-component, Figure 1(e). These behaviors are not unexpected: the estimated values of heading, horizontal component of the acting magnetic field and gyro bias component in the vertical direction must necessarily interact in the attempt to explain the information available to the filter. In other words, the observability conditions can never be too good. However, a safe start in a magnetic homogeneous area [7], and standing still few seconds to carry out the IMU alignment [8] are useful expedients to ensure the validity of the linear approximation of the IMU orientation tracking process. Some disadvantages of the linearization procedure implemented in an EKF are indeed sensitivity to initial conditions, biases in the estimation errors, and critical problems of convergence and filter stability, especially when the sampling interval is too large [28].

The results of the experimental validation support the results of the Monte Carlo study, either in the static case or in dynamic conditions. The importance of implementing sensor fusion algorithms is stressed by the results offered in Table 5. Presently, the reason why the yaw estimates from the Xsens-EKF start to diverge after about 40 s is unknown. The advantage of the compensation of the magnetic disturbances is shown in a situation where the level of contamination is low. It should be noted the importance of providing the filter with some sort of adaptability to the magnetic environment. In this regard, the results of the validation test in static conditions gives rise to several interesting considerations. Since the magnetic variations are quite small, the mechanism of magnetic disturbance compensation is on, with a small value of the process noise standard deviation. This helps making the filter stable; however, its capability of dealing with the magnetic disturbance, when it occurs, is very limited. If, in the effort to deal with the magnetic disturbance, we increase the process noise, the ability to follow the magnetic variations when they occur is remarkable, however the filter may lose track when the magnetic variations are small. On one hand, the injection of process noise is known as a useful recipe to account for mismodeling problems, and even to make the filter response faster [30]; on the other hand, excess process noise is also responsible for less accurate state estimates in benign tracking conditions.

The importance of implementing sensor fusion algorithms is further outlined by the results reported in Table 4. Two further implementations are considered: “only gyro” refers to the case when the EKF is unaided by the accelerometer and the magnetic sensor, and the orientation information is obtained exclusively by time-integrating noisy gyro signals from initial conditions we assume, for simplicity, to be known at the same level of accuracy as in EKF-M_A_ and EKF-M_B_ (we ignore the details of the Xsens’ initialization procedure). This can be obtained by plugging high values of the accelerometer and magnetic sensor measurement noise standard deviation into the filter matrix **R**. On the contrary, the column named “gyro-free” in Table 4 refers to the case when a gyro-free measurement unit is considered. In this case, only the accelerometer and the magnetic sensor are used: their information is combined while solving a specific instance of the Wahba’s problem using the TRIAD algorithm, which is often used to combine measured gravity and Earth magnetic field vectors, for slow motions occurring in magnetically clean environments. Either for the “only gyro” or the “gyro-free” systems, it is worth noting that the stabilization of inclination (roll and pitch) and heading (yaw) is achieved just to a limited extent. In the “only gyro” system, the limited stabilization is a consequence of the random-walk integration of wideband gyro measurement noise, while, in the “gyro-free” system, it is a consequence of the fact that, being a single-frame algorithm for orientation determination, the TRIAD *per se* does not smooth the estimates it produces: even small tremulous movements affecting the accelerometer and any magnetic disturbance in the environment combine their effects to produce noisy orientation estimates. Although the “only gyro” system seems to be almost equivalent to the “gyro-free” system in this particular experimental trial, it must be remembered that random-walk errors tend to grow unbounded over time, while the high-frequency noise affecting the “gyro-free” system is virtually drift-free. However, the “gyro-free” system is completely blind and vulnerable to abrupt changes in the body motion and to magnetic variations in the environment. On the other hand, the EKF is capable of making the best use of the available information, and provides outstanding performance as compared with the “only gyro” and the “gyro-free” systems. Although having a limited ability to overcome magnetic variations, as shown in the static test results, the EKF we have developed may be protected against the effects of body motion using vector selection schemes such as those implemented in [2], which make it adaptive to some extent (**R**-adaptation, or adaptation of the measurement noise covariance matrix **R** is involved in [2]). These schemes are not considered here. An interesting avenue of research will concern how to make the EKF **Q**-adaptive, namely how the process noise covariance matrix **Q** may be changed at run-time to reflect varying magnetic conditions. These considerations stress the importance of developing adaptive EKFs: this is beyond the scope of this paper and it is the subject of our current research. At the present time, we are investigating the rank-condition of observability matrices built from gradients of the Lie derivatives in our research on adaptive EKFs that aims at the compensation of magnetic disturbances in strongly perturbed environments.

The main contribution of this paper concerns the presentation of a method for testing the observability of KFs applied to a problem of orientation determination using inertial/magnetic sensors. Oftentimes, KFs are developed exploiting the considerable freedom in choosing the state and measurement equations, without paying proper consideration to possible problems of observability. This is the first time the observability rank criterion based on Lie derivatives is applied to test the observability of a quaternion-based EKF that is developed for determining the orientation of a rigid body using inertial/magnetic sensors. The formal demonstration of the conditions of observability and, in addition to that, the results of a set of Monte Carlo computer simulations and few experimental trials are offered in support of our approach.

## Figures and Tables

**Table 1.** EKF parameter setting (Monte Carlo simulations).

	**MPE-M_A_**	**MCE-M_A_**	**MPE-M_B_**	**MCE-M_B_**
*σ_g_*, °/s	0.4	0.4	0.4	0.4
*^b^σ_g_*, °/s^2^	0.01	0.01	0.01	0.01
*^b^σ_h_*, mGauss/s	10	1	0	0
*α*, s^−1^	1	1	0	0
*σ_a_*, m*g*	5	5	5	5
*σ_h_*, mGauss	1	1	1	1

**Table 2.** EKF parameter setting (experimental validation).

	**Static**	**Dynamic**
*σ_g_*, °/s	0.4	0.4
*^b^σ_g_*, °/s^2^	0.01	0.01
*^b^σ_h_*, u.a./s (×10^−3^)	10	10
*α*, s^−1^	10	10
*σ_a_*, m*g*	1	5
*σ_h_*, mGauss	1	1

**Table 3.** Monte Carlo simulation results (mean ± SD);

**Condition: MPE**	
**EKF-M_A_**	
Static test	0.93 ± 0.24 ***
Dynamic	1.05 ± 0.24 **

**EKF-M_B_**	
Static test	1.27 ± 0.18 ***
Dynamic	1.53 ± 0.21 **

**Condition: MCE**	
**EKF-M_A_**	
Static test	0.29 ± 0.11
Dynamic	0.32 ± 0.08

**EKF-M_B_**	
Static test	0.22 ± 0.11
Dynamic	0.24 ± 0.15

** SSD at *p* < 0.01;

*** SSD at *p* < 0.001.

**Table 4.** RMSE statistics—EKF *vs.* competing filter implementations.

	**EKF-M_A_**	**EKF-M_B_**	**Only gyro**	**TRIAD**	**Xsens-EKF**
**Roll, °**	0.89	1.02	2.14	2.15	0.92
**Pitch, °**	0.97	0.93	3.85	4.49	0.71
**Yaw, °**	1.14	1.18	7.62	5.81	10.48
**Orientation,°**	1.63	1.69	8.68	7.44	10.60

## Appendix

We prove the conditions under which the 6 × 3 matrix **A** is full rank:

(A1) $$\text{A}=\left[\begin{array}{c} \left[\begin{array}{cc} q_{4}\left[{\text{p}}_{1}\times \right]+\left(\left[{\text{p}}_{1}\times \right]\left[\text{q}\times \right]-2\left[\text{q}\times \right]\left[{\text{p}}_{1}\times \right]\right) & -\left[\text{q}\times \right]{\text{p}}_{1} \end{array}\right]\left[\begin{array}{ccc} {\bar{\text{s}}}_{1} & {\bar{\text{s}}}_{2} & {\bar{\text{s}}}_{3} \end{array}\right]\\ \left[\begin{array}{cc} q_{4}\left[{\text{p}}_{2}\times \right]+\left(\left[{\text{p}}_{2}\times \right]\left[\text{q}\times \right]-2\left[\text{q}\times \right]\left[{\text{p}}_{2}\times \right]\right) & -\left[\text{q}\times \right]{\text{p}}_{2} \end{array}\right]\left[\begin{array}{ccc} {\bar{\text{s}}}_{1} & {\bar{\text{s}}}_{2} & {\bar{\text{s}}}_{3} \end{array}\right] \end{array}\right]$$

Be **p**_1_ = [0 0 *g_z_*]*^T^* and **p**_2_ = [*h_x_* *h_y_* *h_z_*]*^T^*. Let us consider elementary rotations about the three principal axes (roll, pitch and yaw). A script written using the symbolic computation software MAPLE helps proving that minors of order three can always be found with determinant values

(A2) $$\begin{array}{ll} \text{roll}: & \text{cos }\varphi \;h_{x}\cdot g_{z}^{2},\text{ sin }\varphi \;h_{x}\cdot g_{z}^{2},\text{ cos }\varphi \;h_{y}\cdot g_{z}^{2},\text{ sin }\varphi \;h_{y}\cdot g_{z}^{2}\\ \text{pitch}: & \text{cos }\vartheta \;h_{x}\cdot g_{z}^{2},\text{ sin }\vartheta \;h_{x}\cdot g_{z}^{2},\text{ cos }\vartheta \;h_{y}\cdot g_{z}^{2},\text{ sin }\vartheta \;h_{y}\cdot g_{z}^{2}\\ \text{yaw}: & (\text{cos }\psi \;h_{x}+\text{sin }\psi \;h_{y})\;g_{z}^{2},(-\text{sin }\psi \;h_{x}+\text{cos }\psi \;h_{y})\;g_{z}^{2} \end{array}$$

The determinant values cannot be simultaneously zero for any elementary rotation, provided that the local magnetic field has components in the horizontal plane, *i.e.*, *h_x_*, *h_y_* or both are different from zero, condition that can be considered always true, for all practical purposes.

Next, we prove the rank conditions for the 10 × 7 matrix **B**, for which we have:

(A3) $$\Gamma \left(\bar{\text{q}},\text{p}\right)=\left[\begin{array}{cccc} 0 & 0 & 0 & 0\\ 2(-q_{1}p_{3}+q_{3}p_{1}-q_{4}p_{2}) & 2(-q_{2}p_{3}+q_{3}p_{2}+q_{4}p_{1}) & 2(+q_{1}p_{1}+q_{2}p_{2}+q_{3}p_{3}) & 2(-q_{1}p_{2}+q_{2}p_{1}+q_{4}p_{3})\\ 2(+q_{1}p_{2}-q_{2}p_{1}-q_{4}p_{3}) & 2(-q_{1}p_{1}-q_{2}p_{2}-q_{3}p_{3}) & 2(-q_{2}p_{3}+q_{3}p_{2}+q_{4}p_{1}) & 2(-q_{1}p_{3}+q_{3}p_{1}-q_{4}p_{2})\\ 2(+q_{1}p_{3}-q_{3}p_{1}+q_{4}p_{2}) & 2(+q_{2}p_{3}-q_{3}p_{2}-q_{4}p_{1}) & 2(-q_{1}p_{1}-q_{2}p_{2}-q_{3}p_{3}) & 2(+q_{1}p_{2}-q_{2}p_{1}-q_{4}p_{3})\\ 0 & 0 & 0 & 0\\ 2(+q_{1}p_{1}+q_{2}p_{2}+q_{3}p_{3}) & 2(+q_{1}p_{2}-q_{2}p_{1}-q_{4}p_{3}) & 2(+q_{1}p_{3}-q_{3}p_{1}+q_{4}p_{2}) & 2(-q_{2}p_{3}+q_{3}p_{2}+q_{4}p_{1})\\ 2(-q_{1}p_{2}+q_{2}p_{1}+q_{4}p_{3}) & 2(+q_{1}p_{1}+q_{2}p_{2}+q_{3}p_{3}) & 2(+q_{2}p_{3}-q_{3}p_{2}-q_{4}p_{1}) & 2(+q_{1}p_{3}-q_{3}p_{1}+q_{4}p_{2})\\ 2(-q_{1}p_{1}-q_{2}p_{2}-q_{3}p_{3}) & 2(-q_{1}p_{2}+q_{2}p_{1}+q_{4}p_{3}) & 2(-q_{1}p_{3}+q_{3}p_{1}-q_{4}p_{2}) & 2(+q_{2}p_{3}-q_{3}p_{2}-q_{4}p_{1})\\ 0 & 0 & 0 & 0 \end{array}0\right]\;\begin{array}{c} \begin{array}{c} 1\\ 2\\ 3 \end{array}\}\omega_{x}\\ \begin{array}{c} 4\\ 5\\ 6 \end{array}\}\omega_{y}\\ \begin{array}{c} 7\\ 8\\ 9 \end{array}\}\;\omega_{z} \end{array}$$

(A4) $$ϒ\left(\bar{\text{q}}\right)=\left[\begin{array}{ccc} 0 & 0 & 0\\ 2(q_{1}q_{3}+q_{2}q_{4}) & 2(-q_{1}q_{4}+q_{2}q_{3}) & 1-2q_{1}^{2}-2q_{2}^{2}\\ 2(-q_{1}q_{2}+q_{3}q_{4}) & 1-2q_{2}^{2}-2q_{4}^{2} & -2(q_{1}q_{4}+q_{2}q_{3})\\ 2(-q_{1}q_{3}-q_{2}q_{4}) & 2(q_{1}q_{4}-q_{2}q_{3}) & 1-2q_{3}^{2}-2q_{4}^{2}\\ 0 & 0 & 0\\ 1-2q_{2}^{2}-2q_{3}^{2} & 2(q_{1}q_{2}+q_{3}q_{4}) & 2(q_{1}q_{3}-q_{2}q_{4})\\ 2(q_{1}q_{2}-q_{3}q_{4}) & 1-2q_{1}^{2}-2q_{3}^{2} & 2(q_{1}q_{4}+q_{2}q_{3})\\ 1-2q_{1}^{2}-2q_{4}^{2} & -2(q_{1}q_{2}+q_{3}q_{4}) & 2(-q_{1}q_{3}+q_{2}q_{4})\\ 0 & 0 & 0 \end{array}\right]\;\begin{array}{c} \begin{array}{c} 1\\ 2\\ 3 \end{array}\}\;\omega_{x}\\ \begin{array}{c} 4\\ 5\\ 6 \end{array}\}\omega_{y}\\ \begin{array}{c} 7\\ 8\\ 9 \end{array}\}\omega_{z} \end{array}$$

The variables shown right next to the row numbers indicate the control input that is involved in using the corresponding row numbers: when a row is retained in the observability matrix (e.g., the 4^th^ row), the underlying assumption is that the corresponding control input (*i.e.*, *ω_y_*) is non-zero. Note that, for either **Γ** or **ϒ**, rows 2 and 4; 3 and 7; 6 and 8 are equal, apart the sign. Rows 3, 4, 8 (or any other combination of rows that involve, at least, one control input) can be extracted from **ϒ** to form the reduced matrix

(A5) $${ϒ}_{\text{red}}\left(\bar{\text{q}}\right)=\left[\begin{array}{ccc} 2\left(-q_{1}q_{2}+q_{3}q_{4}\right) & 1-2q_{2}^{2}-2q_{4}^{2} & -2\left(q_{1}q_{4}+q_{2}q_{3}\right)\\ 2\left(-q_{1}q_{3}-q_{2}q_{4}\right) & 2\left(q_{1}q_{4}-q_{2}q_{3}\right) & 1-2q_{3}^{2}-2q_{4}^{2}\\ 1-2q_{1}^{2}-2q_{4}^{2} & -2\left(q_{1}q_{2}+q_{3}q_{4}\right) & 2\left(-q_{1}q_{3}+q_{2}q_{4}\right) \end{array}\right]$$

It should be noted that:

(A6) $${ϒ}_{\text{red}}\left(\bar{\text{q}}\right){ϒ}_{\text{red}}^{T}\left(\bar{\text{q}}\right)={\text{I}}_{3\times 3}$$

Hence **ϒ**_red_(**q̄**) is seen to belong to SO(3). It is immediate to conclude that the matrix extracted from **B** by retaining rows 3, 4, 8 and 10, namely:

(A7) $${\text{B}}_{\text{red}}=\left[\begin{array}{ccccccc} 2(+q_{1}p_{2}-q_{2}p_{1}-q_{4}p_{3}) & 2(-q_{1}p_{1}-q_{2}h_{2}-q_{3}p_{3}) & 2(-q_{2}p_{3}+q_{3}p_{2}+q_{4}p_{1}) & 2(-q_{1}p_{3}+q_{3}p_{1}-q_{4}p_{2}) & & & \\ 2(+q_{1}p_{3}-q_{3}p_{1}+q_{4}p_{2}) & 2(+q_{2}p_{3}-q_{3}p_{2}-q_{4}p_{1}) & 2(-q_{1}p_{1}-q_{2}p_{2}-q_{3}p_{3}) & 2(+q_{1}p_{2}-q_{2}p_{1}-q_{4}p_{3}) & & {ϒ}_{\text{red}}\left(\bar{\text{q}}\right) & \\ 2(-q_{1}p_{1}-q_{2}p_{2}-q_{3}p_{3}) & 2(-q_{1}p_{2}+q_{2}p_{1}+q_{4}p_{3}) & 2(-q_{1}p_{3}+q_{3}p_{1}-q_{4}p_{2}) & 2(+q_{2}p_{3}-q_{3}p_{2}-q_{4}p_{1}) & & & \\ 2q_{1} & 2q_{2} & 2q_{3} & 2q_{4} & 0 & 0 & 0 \end{array}\right]$$

is rank 4 if the quaternion is not identically equal to zero, which is true if the quaternion has to represent a valid rotation. We conclude that the motion would be one-dimensional at least, to excite all rows needed for the matrix **B**_red_ to be rank 4; hence the matrix **B** is rank 4 under the same conditions.

By performing Gaussian elimination on the corresponding rows of **O** and eliminating all the elements of the involved columns we obtain:

(A8) $$\text{O}=\left[\begin{array}{ccc} 0_{3\times 4} & 0_{3\times 3} & 0_{3\times 3}\\ 0_{3\times 4} & {}_{ℰ}^{ℬ}\text{C}\left(\bar{\text{q}}\right) & 0_{3\times 3}\\ 0_{3\times 4} & 0_{3\times 3} & {\text{A}}_{11}\\ 0_{3\times 4} & {\text{X}}_{3} & {\text{A}}_{12}\\ {\text{I}}_{4\times 4} & 0_{4\times 3} & 0_{4\times 3}\\ 0_{6\times 4} & 0_{6\times 3} & 0_{6\times 3} \end{array}\right]$$

By performing Gaussian elimination on the rows of the matrix **A**, which is of rank 3, we get:

(A8) $$\text{O}=\left[\begin{array}{ccc} 0_{3\times 4} & 0_{3\times 3} & 0_{3\times 3}\\ 0_{3\times 4} & {}_{ℰ}^{ℬ}\text{C}\left(\bar{\text{q}}\right) & 0_{3\times 3}\\ 0_{3\times 4} & 0_{3\times 3} & {\text{I}}_{3\times 3}\\ 0_{3\times 4} & 0_{3\times 3} & 0_{3\times 3}\\ {\text{I}}_{4\times 4} & 0_{4\times 3} & 0_{4\times 3}\\ 0_{3\times 4} & 0_{3\times 3} & 0_{3\times 3} \end{array}\right]$$

Since a valid DCM is non-singular, we conclude that the observability matrix is full rank when: (a) the magnetic field and the gravity vectors are not collinear; (b) at least one degree of rotational freedom is excited.
